# A predictive model for consciousness recovery of comatose patients after acute brain injury

**DOI:** 10.3389/fnins.2023.1088666

**Published:** 2023-02-08

**Authors:** Liang Zhou, Yuanyi Chen, Ziyuan Liu, Jia You, Siming Chen, Ganzhi Liu, Yang Yu, Jian Wang, Xin Chen

**Affiliations:** ^1^Department of Neurosurgery, Xiangya Hospital of Central South University, Changsha, Hunan, China; ^2^National Clinical Research Center for Geriatric Disorders, Xiangya Hospital, Changsha, Hunan, China; ^3^Central of Stomatology, Xiangya Hospital of Central South University, Changsha, Hunan, China; ^4^College of Intelligence Science and Technology, National University of Defense Technology, Changsha, Hunan, China

**Keywords:** acute brain injury, coma, electroencephalogram (EEG), mismatch negativity (MMN), prognosis, prediction model

## Abstract

**Background:**

Predicting the consciousness recovery for comatose patients with acute brain injury is an important issue. Although some efforts have been made in the study of prognostic assessment methods, it is still unclear which factors can be used to establish model to directly predict the probability of consciousness recovery.

**Objectives:**

We aimed to establish a model using clinical and neuroelectrophysiological indicators to predict consciousness recovery of comatose patients after acute brain injury.

**Methods:**

The clinical data of patients with acute brain injury admitted to the neurosurgical intensive care unit of Xiangya Hospital of Central South University from May 2019 to May 2022, who underwent electroencephalogram (EEG) and auditory mismatch negativity (MMN) examinations within 28 days after coma onset, were collected. The prognosis was assessed by Glasgow Outcome Scale (GOS) at 3 months after coma onset. The least absolute shrinkage and selection operator (LASSO) regression analysis was applied to select the most relevant predictors. We combined Glasgow coma scale (GCS), EEG, and absolute amplitude of MMN at Fz to develop a predictive model using binary logistic regression and then presented by a nomogram. The predictive efficiency of the model was evaluated with AUC and verified by calibration curve. The decision curve analysis (DCA) was used to evaluate the clinical utility of the prediction model.

**Results:**

A total of 116 patients were enrolled for analysis, of which 60 had favorable prognosis (GOS ≥ 3). Five predictors, including GCS (OR = 13.400, *P* < 0.001), absolute amplitude of MMN at Fz site (FzMMNA, OR = 1.855, *P* = 0.038), EEG background activity (OR = 4.309, *P* = 0.023), EEG reactivity (OR = 4.154, *P* = 0.030), and sleep spindles (OR = 4.316, *P* = 0.031), were selected in the model by LASSO and binary logistic regression analysis. This model showed favorable predictive power, with an AUC of 0.939 (95% CI: 0.899–0.979), and calibration. The threshold probability of net benefit was between 5% and 92% in the DCA.

**Conclusion:**

This predictive model for consciousness recovery in patients with acute brain injury is based on a nomogram incorporating GCS, EEG background activity, EEG reactivity, sleep spindles, and FzMMNA, which can be conveniently obtained during hospitalization. It provides a basis for care givers to make subsequent medical decisions.

## Introduction

Acute brain injury, such as, severe traumatic brain injury and intracerebral hemorrhage, results in a heavy burden in low- and middle- income countries for its high mortality and disability rates (Injury, 2019; Krishnamurthi et al., 2020). With the advances of medical care, an increasing number of comatose patients survive. The issue of whether these comatose patients can regain consciousness is significant for doctors and families, especially with regard to the follow-up medical decisions. As we all know, the early intervention is critical for the patients with promising prognosis to regain consciousness. Up to now, there are many methods to assess the prognosis of comatose patients after acute brain injury according to the previous studies (Logi et al., 2011; Ballesteros et al., 2018; Claassen et al., 2019; Monteiro et al., 2021; Romagnosi et al., 2022).

The Glasgow Coma Scale (GCS) score, a reflection of the severity of patients’ condition is currently the most widely used in clinic for its simplicity (Teasdale and Jennett, 1974). Besides, neuroelectrophysiological examinations, including Electroencephalogram (EEG) and event related potentials (ERPs) get more and more attention in clinical practice (Monteiro et al., 2021; Zhou et al., 2021). Among them, EEG plays an important role for outcome prediction in comatose patients (Synek, 1988; Sandroni et al., 2021). EEG background activity, to a large extent, reflects the functional state of the cerebral cortex and subcortex (Estraneo et al., 2020) and EEG reactivity and sleep spindles can provide the additional information about the sensory conduction pathway and thalamic-cortex circuit (Kang et al., 2015; Wang et al., 2022b).

Auditory mismatch negativity (MMN), one of the event-related potentials (ERPs) components, which can be obtained by subtracting the waveform evoked by standard stimuli from the waveform evoked by deviant stimuli, has been increasingly used in comatose patients or prolonged disorders of consciousness in recent years. The presence of MMN indicates that the patient has the ability to voluntarily distinguish and to induce attention orientation to two stimuli, which called pre-attention processing (Alho, 1995). The appearance of MMN suggests a favorable neurological outcome in patients with low responsiveness (Daltrozzo et al., 2007). In addition, the increase of absolute amplitude of MMN was correlated with recovery of consciousness (Wijnen et al., 2007; Zhou et al., 2021).

However, it is an important but still unclear clinical issue which assessment indicators to choose to achieve the best predictive performance. In this study, we used least absolute shrinkage and selection operator (LASSO) and binary logistic regression analysis methods to establish a prediction model, which made the probability of the consciousness recovery for comatose patients visualized by nomogram. In this way, it can provide a better basis for clinical practice.

## Materials and methods

### Patients

**FIGURE 1:**
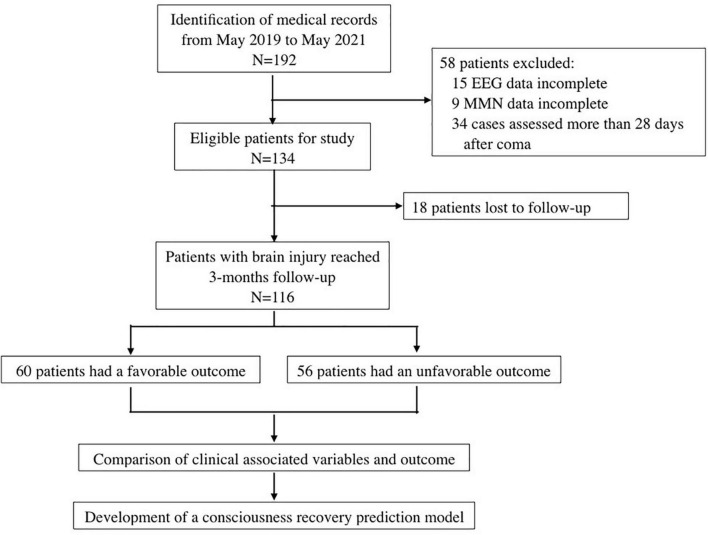
Flow chart of this study.

We retrospectively reviewed the records of patients with severe brain injury who were admitted to the Neurosurgical Intensive Care Unit of Central South University Xiangya Hospital from May 2019 to May 2022. The inclusion criteria were as follows: (1) craniocerebral injury caused by traumatic brain injury and intracerebral hemorrhage and confirmed by computed tomography or magnetic resonance imaging; (2) GCS ≤ 8 at the time of electrophysiological assessment; (3) age ≥ 18 years old; and (4) EEG and auditory MMN examinations performed within 28 days from coma onset. The exclusion criteria were: (1) pre-existing neurological diseases; (2) known hearing impairment; (3) received sedatives and/or muscle relaxants, except for dexmedetomidine or low dose of midazolam (≤ 0.02 mg/kg/h) (Riker et al., 2009), during the course of EEG and MMN examinations; and (4) incomplete clinical data. The charts of patients enrollment process of this study was in Figure 1.

### MMN paradigm

**FIGURE 2:**
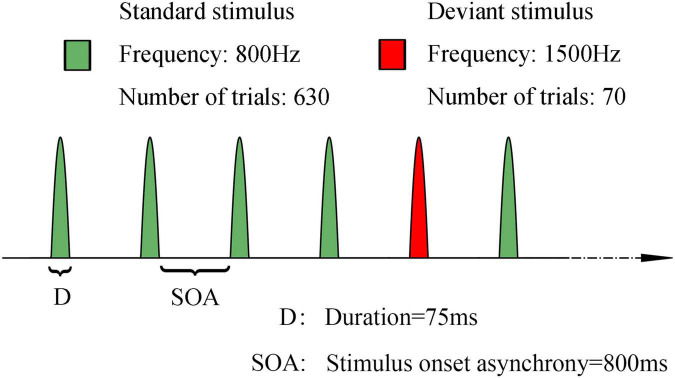
Schematic representation of the stimulus paradigm of mismatch negativity. Green: standard stimuli, 800 Hz, 630 trials. Red: deviant stimuli, 1500 Hz, 70 trials. Duration of each sound stimuli is 75 ms. SOA: 800 ms. The sound stimuli were continuously and pseudorandomly presented, although there were at least three standard stimuli between two consecutive deviants.

We used a classical oddball auditory paradigm to elicit auditory MMN. It comprised two types of pure sound with different frequencies: 800 Hz and 1,500 Hz for the standard and deviant stimuli, respectively. In this paradigm, 700 pure sound stimuli (comprising 90% standard stimuli and 10% deviant stimuli, 80 dB sound pressure level, lasting for 75 ms) with a stimulus onset asynchrony of 800 ms were presented to every patient to elicit the MMN response. The sound stimuli were continuously and pseudorandomly presented, although there were at least three standard stimuli between two consecutive deviants. The sound was delivered through headphones. The whole examination lasted about 11 min. Schematic diagram of MMN paradigm was presented in Figure 2.

### MMN data acquisition and analysis

Scalp MMN examinations were performed at the patients’ besides, while they were free from visible body shaking. Data were recorded at four electrodes (F3, F4, Fz, and Cz) according to the 10–20 international system using a Rinjie medical event-related potentiometer (WJ-IA, Guangzhou, China). The impedance of all electrodes was kept below 5 KΩ, and the sampling rate was 1,024 Hz with an online 1–100 Hz bandpass filter. Data were referenced with the mean potential at electrodes A1 and A2.

Raw ERP data with amplitudes exceeding 100 μV were automatically rejected, thus eliminating eye movements and other artifacts. Subsequently, the standard and deviant responses were averaged by extracting the data of 100 ms before each stimulus onset and 500 ms after the stimulus; the former was primarily used for baseline correction. After that, a 3–30 Hz bandpass filter is applied and the MMN can be obtained by subtracting the waveform evoked by standard stimuli from the waveform evoked by deviant stimuli. Finally, ERP components such as N1 and MMN were presented and calculated by an automatic algorithm.

The criteria for identifying MMN components were: (1) Considering that N1 is one of the representative indicators of the auditory gating system and the information stream among the auditory cortex areas is directed from the core area to the band area and then to the sub-band area. Meanwhile, the core area and band area are the main generators of the standard N1 and deviation N1 components, respectively (Jones, 2002). We considered that MMN was not present only if the standard and deviation N1 were both evoked (Rosburg, 2019); (2) The largest negative waves of averaged difference waveforms in the latency interval between 100 and 300 ms were considered to represent the presence of MMN components (Grimm et al., 2011; Wang et al., 2018); (3) Considering that some of the patients underwent unilateral or bilateral decompressive craniectomy and MMN had maximal amplitude at Fz (Wang et al., 2018), we mainly investigated the absolute amplitude of MMN at the Fz site in this study.

### EEG data acquisition and analysis

Continuous digital EEG monitoring (SOLAR electroencephalogram acquisition system, Beijing, China) was routinely performed and lasted for over 24 h in the study, with 16 electrodes (FP1, FP2, F3, F4, C3, C4, P3, P4, 01, 02, F7, F8, T3, T4, T5, and T6) placed according to the 10–20 international system. Video was simultaneously recorded to identify clinical events and artifacts. All EEG data were analyzed by two certified neurophysiologists.

Electroencephalogram background activity was classified into spindle coma, alpha coma, and five other categories as follows (Synek, 1988; Estraneo et al., 2020): (1) normal EEG activity, with a predominant posterior alpha rhythm and anterior-posterior gradient (APG), without focal or hemispheric slowing or epileptiform abnormalities; (2) mildly abnormal (MiA) EEG, characterized by predominant posterior theta activity (> 20 μV), symmetric or not, with frequent (10–49% of recording) posterior alpha rhythms; (3) moderately abnormal (MoA) EEG, characterized by predominant posterior theta activity (> 20 μV), symmetric or not, poorly organized APG, with rare (< 1% of recording) or occasional (1–9% of recording) posterior alpha rhythms; (4) diffuse slowing (DS), defined as EEG background activity with predominant diffuse theta or theta/delta rhythms with an amplitude > 20 μV, without APG; (5) low voltage (LV) EEG, defined as predominant EEG activity (theta or delta) < 20 μV over most brain regions.

Sleep spindles were defined as 10–15 Hz bursts, lasting 0.5–2 s, that were best seen in the central channel (Rasch and Born, 2013). Sleep spindles that were not clearly detectable were considered as absent.

EEG-R was performed as follows (Admiraal et al., 2018): (1) pain stimulus: the patient’s nail bed was pressed using a pen for at least 5 s. There were three repeats at each side with each pressing separated by a 5-min interval. (2) Sound stimulus: loud claps were performed for at least 5 s near the patient’s ear on one side. There were three repeats at each side with each clapping separated by a 5-min interval. The presence of EEG-R was defined as stable and repeatable (at least twice) changes in amplitude or frequency in the EEG, which were visible with the naked eye, except for muscle and eye blink artifacts within at least one stimulus type.

The percent of alpha variability (PAV) was determined by visual inspection of three points on the PA histogram: the PA baseline, the PA peak value, and the PA trough value that most directly follows the peak. The PA baseline was deemed to the mean PA occurring during the 4 h prior to a peak in PA. The PAV was calculated using the following formula: (Peak PA–Trough PA)/(Peak PA + Trough PA). Then, the PAV were divided into four levels, which were poor (level 1, < 2%), fair (level 2, 2–10%), good (level 3, 10–15%), and excellent (level 4, > 15%), respectively (Vespa et al., 1997). PAV of two bipolar channels of each patients were calculated using bilateral frontoparietal, which were (F3–P3) and (F4–P4) (Friberg et al., 2013). Besides, the global PAV scores were evaluated as the mean of the two hemispheric PAV values (Hebb et al., 2007).

### Prognosis assessment

The prognosis of patients was determined by telephone follow-up 3 months after coma onset. The Glasgow Outcome Scale (GOS) ranging from 1 to 5 (1, dead; 2, vegetative state or minimally conscious state; 3, able to follow commands but unable to live independently; 4, able to live independently but unable to return to work or school; or 5, able to return to work or school) (Jennett et al., 1981), was used to evaluate prognosis. GOS scores 1–2 were defined as unfavorable prognosis (no recovery of consciousness), and GOS scores 3–5 were defined as favorable prognosis (recovery of consciousness).

### Predictive variables

The variables including sex, age, etiology, pupillary light reflex, GCS, absolute amplitude of N1 at electrode Fz (FzN1A), absolute amplitude of MMN at electrode Fz (FzMMNA), EEG background activity, sleep spindles, EEG-R, and PAV, which were generally considered to be associated with the prognosis of comatose patients. These 11 factors were preliminary screened, using the least absolute shrinkage and selection operator (LASSO) analysis. LASSO analysis was conducted with R software (version 4.2.1).

### Statistical analysis

In this study, continuous and categorical variables were expressed by the mean value ± standard deviation or median (interquartile range, IQR) and the frequency (percentage), respectively.

The binary logistic regression model was used for multivariate analysis of wakefulness in comatose patients. A nomogram for the predictive model was developed based on the results of the binary logistic analysis. The calibration of the nomogram was used for internal validation by the 100 bootstrap resampling procedure. In addition, the predictive efficiency of the nomogram was quantified with area under the curve (AUC) of receiver operating characteristic curve (ROC). Furthermore, the clinical utility of the nomogram was assessed by the decision curve analysis (DCA). Statistical analysis was conducted with R software (version 4.2.1). For all tests, statistically significance was set at *P* < 0.05.

## Results

### Patients

A total of 116 patients, including 29 females and 87 males, were enrolled in our study. There were 73 cases of traumatic brain injury and 43 cases of intracerebral hemorrhage. The mean age was 51.8 ± 16.4 years old, and the EEG and MMN recordings took place on average 14 (10.0, 21.3) days after coma onset. The most frequent pattern of predominant EEG activity was diffuse slowing (DS), followed by moderately abnormal (MoA); 4 patients showed a spindle coma pattern and 2 showed an alpha coma pattern. Of 116 patients, 60 had favorable outcomes and 56 had unfavorable outcomes. Additionally, components N1 and MMN were absent in 19 patients. And in our study, there were no patients with normal or alpha coma EEG background activity. Compared with the unfavorable prognosis group, the percentage of the GCS 6–8 scores, presence of EEG-R and sleep spindles, and better EEG background activity in favorable prognosis group were significantly higher. Furthermore, the mean amplitude of FzMMNA in favorable prognosis group was higher than the other group. These baseline characteristics were shown in Table 1.

**TABLE 1:** Demographic and clinical characteristics of the patients in favorable and unfavorable prognosis groups.

Factors	Favorable prognosis (*n* = 60)	Unfavorable prognosis (*n* = 56)
**Sex [*n* (%)]**		
Male	49 (56.3)	38 (43.7)
Female	11 (37.9)	18 (62.1)
**Etiology [*n* (%)]**		
Traumatic brain injury	37 (50.7)	36 (49.3)
Intracerebral hemorrhage	23 (53.5)	20 (46.5)
Pupillary light reflex* [*n* (%)]		
Presence	58 (56.3)	45 (43.7)
Absence	2 (15.4)	11 (84.6)
**GCS [*n* (%)]**		
3–5	15 (23.8)	48 (76.2)
6–8	45 (84.9)	8 (15.1)
**EEG background activity [*n* (%)]**		
Normal/MiA/MoA/Spindle coma	48 (71.6)	19 (28.4)
DS/LV/Alpha coma	12 (24.5)	37 (75.5)
**EEG-R [*n* (%)]**		
Presence	32 (78.0)	9 (22.0)
Absence	28 (37.3)	47 (62.7)
**Sleep spindles [*n* (%)]**		
Presence	39 (86.7)	6 (13.3)
Absence	21 (29.6)	50 (70.4)
**PAV [*n* (%)]**		
Level 1	13 (40.6)	19 (59.4)
Level 2	25 (46.3)	29 (53.7)
Level 3	22 (73.3)	8 (26.7)
Age (years, $\bar{x}$ ± *s*)	50.4 ± 17.1	53.4 ± 15.6
FzN1A (μV, $\bar{x}$ ± *s*)	1.4 ± 1.1	0.9 ± 1.1
FzMMNA (μV, $\bar{x}$ ± *s*)	2.1 ± 1.2	1.2 ± 0.8

*Only if pupillary light reflex disappeared bilaterally was considered as absent. FzN1L, the peak latency of N1 at electrode Fz; FzN1A, the absolute amplitude of N1 at electrode Fz; FzMMNL, the peak latency of MMN at electrode Fz; FzMMNA, the absolute amplitude of MMN at electrode Fz; EEG-R, electroencephalogramactivity; MiA, mildly abnormal; MoA, moderately abnormal; DS, diffuse slowing; LV, low voltage; PAV, percent alpha variability.

### Predictors selection

**FIGURE 3:**
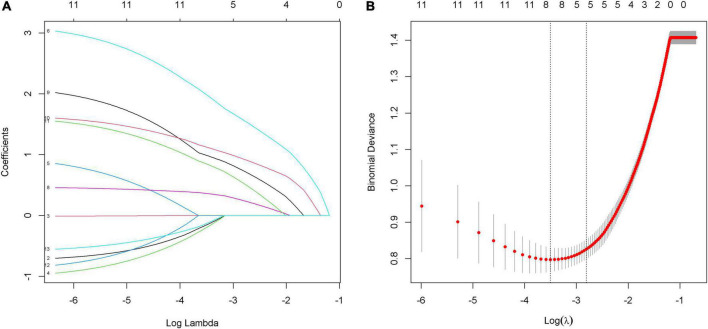
Predictors selection by using the least absolute shrinkage and selection operator (LASSO) model. **(A)** LASSO coefficient profiles of 11 alternative factors. Each curve represents a factor. Since PAV is a triadic variable, it was divided into three variables when analyzed in R language software. As a result, there are thirteen curves shown in the figure. **(B)** Tuning parameter (λ) selection in the LASSO model using 10-fold cross-validation *via* minimum criteria. The red dots in the figure indicate the binomial deviance corresponding to each lambda. The two dashed lines represent two special lambda values, min and 1 s, respectively. The 1 s corresponds to the optimal lambda value of a model with excellent performance and the smallest number of independent variables. Finally, combined with figure **(A)**, five factors (GCS, FzMMNA, EEG background activity, EEG-R, and sleep spindles) were selected into the following binary logistic regression analysis.

Eleven factors, sex, age, etiology, pupillary light reflex, GCS, FzN1A, FzMMNA, EEG background activity, EEG-R, sleep spindles, and PAV, were extracted from demographic characteristics, clinical features, and EEG and MMN examination related indictors. Of these, 5 factors, GCS, FzMMNA, EEG background activity, EEG-R, and sleep spindles, were selected as potential predictors by LASSO analysis (Figure 3). It should be mentioned that Lasso analysis did not provide *p*-value in R language. Furthermore, these 5 predictors have been shown to be significantly associated with wakefulness of comatose patients after acute brain injury by binary logistic regression analysis (*P* < 0.05). Additionally, the results were expressed as odd ratio (95% confidence interval) for GCS [13.400 (3.976, 54.478)], FzMMNA (μV) [1.855 (1.085, 3.510)], EEG background activity [4.309 (1.262, 16.251)], EEG-R [4.154 (1.177, 15.965)], and sleep spindles [4.316 (1.169, 17.253)]. The Positive Predictive Value (PPV) and Negative Predictive Value (NPV) of EEG-R were 78.0 and 62.7%, respectively. The results of binary logistic regression was shown in Table 2.

**TABLE 2:** Binary logistic regression analysis of the predictors of consciousness recovery in comatose patients.

Predictors	OR (95% CI)	*P*-values
GCS	13.400 (3.976, 54.478)	<0.001
FzMMNA	1.855 (1.085, 3.510)	0.038
EEG background activity	4.309 (1.262, 16.251)	0.023
EEG-R	5.154 (1.177, 15.965)	0.030
Sleep spindles	4.316 (1.169, 17.253)	0.031

OR, odd ratio; CI, confidence interval; FzMMNA, the absolute amplitude of MMN at electrode Fz; EEG-R, electroencephalogram activity.

### Development of a prediction nomogram

**FIGURE 4:**
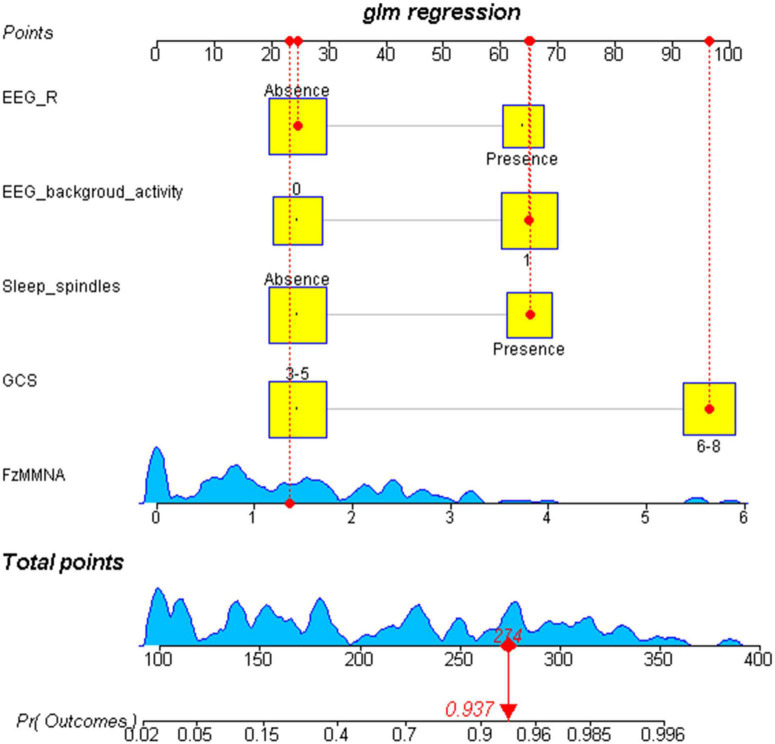
Visualization nomogram to predict the consciousness recovery of comatose patients after acute brain injury. The total score of each variable and the corresponding probability of consciousness recovery of comatose patients can be visualized in the nomogram. For example, a case is shown in the nomogram (red). This case represents a comatose patient with GCS scores of 8, the EEG-R is absent, the sleep spindles is present, the EEG background activity at level 3, and the FzMMNA is 1.42 μV. In consideration of these five variables, the total score for this patient was 274, corresponding to a probability of regaining consciousness of 0.937. Number 0 in EEG background activity refers to diffuse slowing (DS), low voltage (LV), and Alpha coma. Number 1 in EEG background activity refers to normal, mildly abnormal (MiA), moderately abnormal (MoA), and spindle coma.

After the binary logistic regression analysis, the independent predictors including GCS, FzMMNA, EEG background activity, EEG-R, and sleep spindles were used for developing a prediction nomogram. Nomogram, like a scoring system, was established based on the odd ratio values. The score of each predictor can be clearly seen in this system. In addition, the sum of scores and corresponding probabilities for the consciousness recovery of each comatose patient after acute brain injury can be effectively estimated and visualized in the nomogram (Figure 4).

### Validation of the nomogram

**FIGURE 5:**
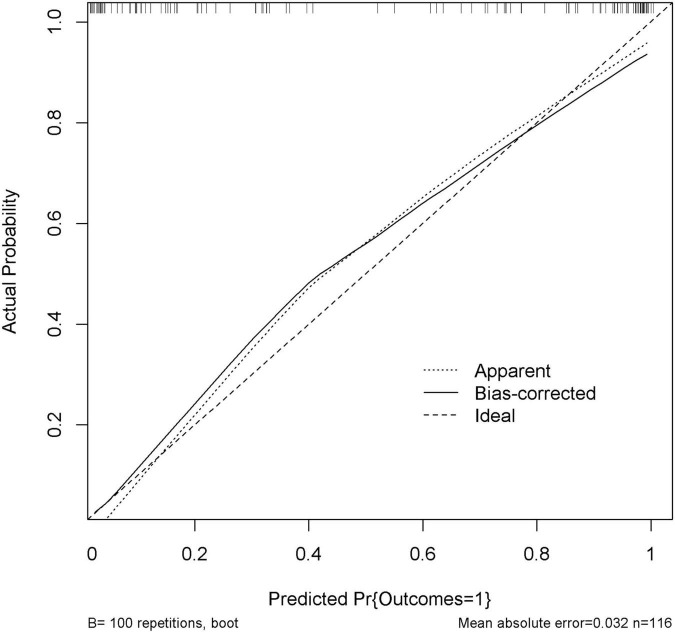
Calibration curve of the nomogram. Internal validation was performed using the 100 bootstrap resampling method. The predicted probability and the actual probability are presented by the *X* and *Y* axes, respectively.

**FIGURE 6:**
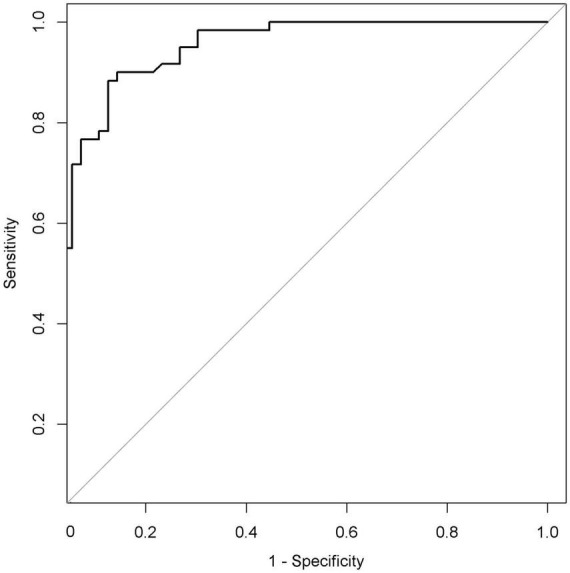
Receiver operating characteristic curve (ROC) of the nomogram. The AUC of the nomogram was 0.939 (95% CI: 0.899–0.979). AUC, area under the curve.

The performance of nomogram was internally validated by using the 100 bootstrap resampling method, in which the model development cohort (original sample) served as the validation set, and each resampled sample cohort served as the training set. The calibration curve (Figure 5) showed a high consistency between the observed and predicted values in the probability of the consciousness recovery of comatose patients after acute brain injury. Besides, the AUC [0.939 (95% CI: 0.899–0.979)] was obtained for evaluating the accuracy of this predictive model by ROC (Figure 6).

### Clinical utility of the nomogram

**FIGURE 7:**
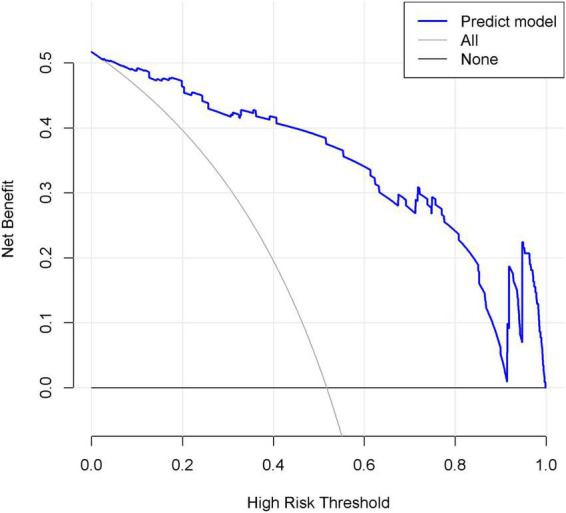
Decision curve analysis (DCA) of the nomogram. *X*-axis and *y*-axis represent threshold probability and net benefit, respectively. When the threshold probability value of the predictive model is between 0.05 and 0.92, the patients will obtain the corresponding net benefit.

In the DCA shown in Figure 7, the threshold probability of the nomogram ranged from 5 to 92%, which indicated a wide range of clinical utility. In other words, the nomogram will achieve the net benefit in varying degrees when the threshold probability value of the predictive model is between 0.05 and 0.92. Moreover, the reason for the fluctuation at the end of the prediction model curve is mainly considered as the sample size is not large enough.

## Discussion

Multimodal monitoring can be applied in the assessment of consciousness recovery of comatose patients after acute brain injury. Although some efforts have been made in resolving this issue, it is still unclear which factors can be used to establish model to directly predict the probability of patients’ consciousness recovery. In fact, the results of the comprehensive assessment will affect doctors’ medical decisions determining final outcome of patients to a great extent. In this study, clinical characteristics and parameters derived from electrophysiological examinations, including GCS, FzMMNA and EEG background activity, EEG-R, and sleep spindles have been selected in the predictive model by LASSO and binary logistic regression analysis. By intuitively predicting the consciousness recovery of comatose patients after acute brain injury, this predictive model can identify patients with relatively favorable prognosis at early stages and allows active clinical intervention.

Glasgow coma scale, a clinical behavioral scale, is easy to perform and widely used in the intensive care unit and often included in prognostic models. Our study found that GCS correlated with the outcomes of comatose patients (*P* < 0.001); the findings are in accordance with those in previous studies (Emami et al., 2016; Nik et al., 2018).

Electroencephalogram is the result of recording the spontaneous electrophysiological activity emitted by cortical vertebral cells of the human brain. Three EEG related indicators in the predictive model can be applied to assess the brain injury condition of comatose patients from different layers. The EEG background activity reflects the electrical activity of the patient’s brain and it’s a clue to how active the brain is. EEG background activity was classified into two categories based on predominant frequency and amplitude (Wang et al., 2022a), and proved to be associated with the prognosis of comatose patients. At baseline, the two prognostic groups differed significantly for predominant EEG background activity, since poor EEG patterns was more frequently observed in patients in unfavorable group, whereas mildly and moderate abnormal EEG activities in another group. Besides, two special EEG coma patterns, alpha and spindle coma were also included. When a prominent generalized, often frontally predominant, non-reactive alpha frequency activity constitute the principal EEG features in comatose patients, the patterns are referred to as alpha coma (Nuwer, 2021). And this kind of pattern often imply a poor prognosis, because its appearance suggests either the inputs from the thalamus or the neural filtering networks of the cortex are disturbed, or both are damaged. Spindle coma is an electroclinical entity that has been used to describe an EEG pattern of “sleep-like” activity in comatose patients (Emidio et al., 2019). Although, the spindle coma is usually resulted from a pontomesencephalic junction lesion (Husain, 2006), it is associated with favorable outcome in most situations, according to previous studies (Cologan et al., 2013; Rasch and Born, 2013). For the reason that existence of spindles often indicates the residual function of thalamus and thalamic-cortex circuit. On the contrary, the absence of spindles in comatose patients with acute brain injury results from the interruption of either the ascending reticular thalamocortical pathway or of the thalamocortical loops, which may result in difficulty in regaining consciousness.

EEG-R is defined as the diffuse, transient, and repeatable changes in electroencephalogram activity (amplitude and/or frequency) following environmental stimuli, such as pain or sound (Azabou et al., 2018a). The presence of EEG-R suggests the integrity of the peripheral sensory pathways, brainstem, subcortical structure, and cerebral cortex, and lack of EEG-R may indicate severe dysfunction in any of the aforementioned structures, tampering the cortical activity secondary to external stimuli (Altwegg-Boussac et al., 2017). Our study found that EEG-R was an independent predictive factor for outcome in comatose patients with acute brain injury. In addition, we noticed that EEG-R had a PPV of 78.0% and a relatively low NPV of 62.7%. We propose that this finding might be attributed to the fact that the results were evaluated with the naked eye because the stimulation protocol in EEG-R has not been uniformly standardized. Accordingly, subjectivity cannot be ruled out when assessing the presence of EEG-R. In other words, the absence of EEG-R, as judged by the examiner, does not necessarily reflect the real brain functional status of the patients. Thus, further studies are warranted to better standardize and clarify the EEG-R protocol.

Mismatch negativity is the maximal negative wave presented after standard and deviant stimuli during the latency of 100–250 ms. In patients with disorders of consciousness, the latency can be up to 300 ms (Wang et al., 2018). MMN indicates the cortical function of differentiation and orientation to different sound stimuli, or so-called pre-attention processing. MMN originates from the auditory cortex in the Heschl’s gyri and fronto-central area, and generally has a maximal amplitude at the Fz site (Pakarinen et al., 2007; Wang et al., 2020). Besides, MMN can also be evoked in patients under sedation (Azabou et al., 2018b), anesthesia (Kenemans and Kahkonen, 2011), sleep (Chennu and Bekinschtein, 2012), and disorders of consciousness (Morlet and Fischer, 2014; Wang et al., 2020). Previous studies have demonstrated a correlation between MMN and favorable outcomes, yet few studies have incorporated the MMN amplitude into quantitative analysis. Subjective factors can easily be mixed into the interpretation of MMN, and it is difficult to apply in clinical practice (Morlet and Fischer, 2014; Schall, 2016). Therefore, we investigated the relationship between FzMMNA and patient’s outcomes and found that FzMMNA could be a valuable indicator to predict consciousness recovery of comatose patients. It is worth mentioning that patients who regained consciousness had higher MMN amplitudes than patients who did not. A higher MMN amplitude suggests an increase in alertness levels, which is crucial for detecting changes in the environment and adapting behavior. Indeed, the MMN amplitude is also possibly associated with the binding of the glutamic acid and the N-methyl-D-aspartate receptors, which play an important role in modulating MMN-indexed auditory discrimination (Impey et al., 2016); this may be the reason why MMN amplitude increased in DoC patients who regained consciousness after tDCS treatment (Wang et al., 2020).

In this study, age, coma etiology, pupillary light reflex as well as PAV were not associated significantly with prognosis in our study. This result may be attributed to the selection bias and limited sample size. Certainly, further studies with a larger sample size are needed for verification.

In deed, 5 indicators in our predictive model evaluated the brain function of the comatose patients after acute brain injury from different dimensions and achieved a high accuracy (AUC = 0.939). As long as conditions permit, we should use as many tools as possible to predict the prognosis of patients synthetically in clinical practice.

## Limitations

There are three major limitations in our study. Firstly, the sample size of this study was not large enough to support the selection of more factors into the model. As we all know, the prognosis assessment of comatose patients after brain injury requires as many monitoring methods as possible to achieve a more accurate and stable prediction. Secondly, taking into account the medical safety and clinical needs of patients, it was impossible to eliminate prognosis in clinical work without using any sedative drugs when EEG and MMN examinations were performed; thus, we included patients treated with dexmedetomidine, which is commonly used in our ward for sedation. Thirdly, most of the patients in our study underwent neurosurgery, while a small number did not. This difference may affect the outcome of patients. These three limitations may affect the results of this study.

## Conclusion

In summary, our study develops a promising prediction model with a wide range of clinical utility for the consciousness recovery of comatose patients after acute brain injury. Besides, considering the spread of clinical practice, it is critical that, as a clinical tool, the predictors in the model can be conveniently obtained at the patients’ bedsides, which will provide important reference value for clinicians. Certainly, further prospective multimodal monitoring to predict the consciousness recovery of comatose patients after brain injury study is needed for the establishment of prediction model.

## Data availability statement

The raw data supporting the conclusions of this article will be made available by the authors, without undue reservation.

## Ethics statement

The studies involving human participants were reviewed and approved by the Medical Ethics Committee of Xiangya Hospital of Central South University ethical approval number (202210230). Written informed consent for participation was not required for this study in accordance with the national legislation and the institutional requirements.

## Author contributions

LZ, JW, YC, and XC contributed to the consult literature materials and design the study. LZ and JW acquired the data. LZ, YY, and XC analyze the data. LZ, SC, and GL prepared the tables and figures. LZ, JW, and ZL were major contributors in writing the manuscript. JY, YC, and XC revised the manuscript and made the final version of the manuscript. All authors read and approved the final manuscript.
